# Fractures of the Inferior Maxilla

**Published:** 1858-02

**Authors:** O. C. Gibbs

**Affiliations:** Frewsburg, Chautauque Co., N. Y.


ART. III.—*Fractures of the Inferior Maxilla.* By O. C. GIBBS, M. D., Frewsburg, Chautauque Co., N. Y.

The cases of fracture of the inferior maxilla, reported in the December number of your Journal by Prof. F. H. Hamilton, bring to mind a case of unusually extensive fracture of the inferior maxilla, which came under my observation some months since. The case may be not altogether unworthy of record.

April 5th, 1856, I was called to see Mr. McC., an Irish laborer, aged about 40 years. He had received a severe blow upon the left side of the inferior maxilla from a handspike, the motive power being a heavy log. He was assisting in rolling a large log upon skids, when it was carelessly rolled off the end of the skids, dropping about two feet; in the fall it struck the lower end of his handspike, knocking the end which he held from his hands, which end forcibly struck him on the side of the face and laid him senseless upon the ground.

As the patient resided about twelve miles away, I did not see him until several hours after the accident. Consciousness soon returned, and on my arrival I found the soft parts much tumefied, which, with the deformity resulting from the fractures soon to be mentioned, gave the patient a rather singular expression.

On examination, the inferior maxilla was found broken in *three* distinct places. The teeth in the upper jaw were entire, and the fragments of the bone were carefully moulded to an exact adaptation to the upper maxilla, with the design of making it subserve the purpose of an efficient splint to hold in place the fragments of the broken bone. In consequence of the great tumefaction, it was thought advisable to depart from the ordinary manner of applying bandages,—it was thought that the pressure of the bandages upon the injured side of the face would, in consequence of its swollen condition, displace the fragments of the jaw inward. To prevent this, a splint, somewhat of an horse-shoe shape, was fitted to the inferior surface of the fractured bone, projecting sufficiently beyond it on the sides to guard against lateral pressure. This splint was well padded and applied, and held in place by suitable bandages fastened to a cap of firm cloth fitted to the head. The patient was supported by liquid nourishment, conveyed to the mouth through a space left by an absent tooth. The fracture healed readily and without deformity. Suppuration of the bruised parts took place to some extent, and one tooth and a spicula of bone were removed before perfect soundness was restored.

In the same number of the Journal referred to above, there is a passage in Dr. Nichols' report of Prof. Flint's Clinical Lectures, in regard to which I wish to add a thought. I quote the passage referred to: "In the patient who is now convalescing from typhoid fever, a somewhat *unusual*, and at first view, *strange measure*, was put in practice several days, since during the height of the fever. The patient had presented for some time previous to this, distension of the abdomen, with tympanites, accompanied with frequent liquid evacuations passed in bed. The patient was actively delirious. His pulse was not small, nor was it much accelerated. Prof. F. regarding the diarrhœa as an effort of the system to relieve the intestines of their contents, directed a *saline cathartic* to be given: magnesiæ sulph., ℥j, every two hours until the evening, this being about noon. The following morning, the nurse stated that two doses of the above medicine had been taken, and a copious dejection occurred during the night. Since then, and for three days thereafter, no dejection was observed. There was less distension of the abdomen, and also less tympanites, and in improvement in the condition of the patient generally." This treatment, with me at least, is not altogether new. If Prof. Flint will, *in like cases*, add from fifteen to twenty drops of *elixir vitriol* to each dose of the saline solution, he will have a combination, which, from my rather limited experience, he will seldom have occasion to repent the administration, under circumstances as detailed.

